# Immediate and long-term consequences of COVID-19 infections for the development of neurological disease

**DOI:** 10.1186/s13195-020-00640-3

**Published:** 2020-06-04

**Authors:** Michael T. Heneka, Douglas Golenbock, Eicke Latz, Dave Morgan, Robert Brown

**Affiliations:** 1 Department of Neurodegenerative Disease and Geriatric Psychiatry, University of Bonn, Bonn, Germany; 2 German Center for Neurodegenerative Disease, Bonn, Germany; 3 Division of Infectious Diseases and Immunology, University of Massachusetts Medical School, Worcester, MA 01605 USA; 4 Institute for Innate Immunity, University of Bonn, Bonn, Germany; 5 Translational Neuroscience, College of Human Medicine, Michigan State University, Grand Rapids, USA; 6 Department of Neurology, University of Massachusetts Medical School, Worcester, MA 01605 USA

**Keywords:** Systemic inflammation, NLRP3 inflammasome, Cytokine, Cognition, Neurodegeneration, Neuroinflammation, Decline

## Abstract

Increasing evidence suggests that infection with Sars-CoV-2 causes neurological deficits in a substantial proportion of affected patients. While these symptoms arise acutely during the course of infection, less is known about the possible long-term consequences for the brain. Severely affected COVID-19 cases experience high levels of proinflammatory cytokines and acute respiratory dysfunction and often require assisted ventilation. All these factors have been suggested to cause cognitive decline. Pathogenetically, this may result from direct negative effects of the immune reaction, acceleration or aggravation of pre-existing cognitive deficits, or de novo induction of a neurodegenerative disease. This article summarizes the current understanding of neurological symptoms of COVID-19 and hypothesizes that affected patients may be at higher risk of developing cognitive decline after overcoming the primary COVID-19 infection. A structured prospective evaluation should analyze the likelihood, time course, and severity of cognitive impairment following the COVID-19 pandemic.

## Background

The COVID-19 pandemic represents an unprecedented immediate but also persisting threat to our health care systems. The immediate urgency is clear, as the total number of affected patients exceeds most of the health care system capacities worldwide, particularly for the treatment of patients requiring assisted ventilation. Less immediately apparent, but potentially very significant are long-term consequences of COVID-19 infections.

During the acute phase of COVID-19 infection, about 36% of cases develop neurological symptoms of which 25% can be attributed to the direct involvement of the central nervous system. The main symptoms include but are not restricted to dizziness, headache, impaired consciousness, and seizure [1]. Patients who show neurological symptoms included cases with or without pre-existing neurological disorders [2]. While on intensive care units, patients showed agitation, confusion, and corticospinal tract signs such as enhanced tendon reflexes and clonus. In mild to moderate disease cases, patients reported olfactory (85.6%) and gustatory (88.0%) dysfunctions. Importantly, in about 11% of patients, anosmia occurred prior to any other clinical symptoms [3]. COVID-19 can further lead to changes of coagulation and, in particular, to inflammation-induced disseminated intravascular coagulation (DIC). Together with endothelial dysfunction, DIC can cause cerebrovascular ischemia even in young patients, many of whom suffer from large vessel ischemic stroke [4, 5]. Overall, this may also be influenced by the severity of the COVID-19 infection, as 5.7% of the severe cases suffered from ischemic stroke [1] or had pre-existing vascular risk factors, especially in the elderly, in whom ischemic stroke rather occurred as a delayed complication [6, 7]. Additionally, as sub-acute signs that occurred 3–10 days after the development of Covid-19 symptoms, Guillain-Barré syndrome [8] and Miller-Fisher syndrome [9] cases have been reported. Also, clinically striking are cases of Kawaski-like multisystem inflammatory syndromes now being recognized in children and teenagers.

## Main text

There are at least four possible pathogenic mechanisms that may account for the detrimental effect of COVID-19 on the CNS: (1) direct viral encephalitis, (2) systemic inflammation, (3) peripheral organ dysfunction (liver, kidney, lung), and (4) cerebrovascular changes. In most cases, however, neurological manifestations of COVID-19 may arise from a combination of the above.

Any one or a combination of these mechanisms put COVID-19 survivors at risk for developing long-term neurological consequences, either by aggravating a pre-existing neurological disorder or by initiating a new disorder. This concern is supported by findings that show that one third of patients at the time of discharge have evidence of cognitive impairment and motor deficits [2]. This is particularly relevant because overall COVID-19 clinically affects the elderly most severely [10]. There is a large overlap of the age range when people typically develop neurodegenerative or cerebrovascular disease and the age of risk for the most several COVID-19 infections. This overlap argues that there is a compelling need for prospective neurological surveillance and care.

COVID-19 is associated with a severe innate immune response and sustained rise of systemic cytokine levels. Importantly, this innate immune response has been suggested to drive and predict mortality and severity [11]. Cytokines and related inflammatory mediators found to be elevated include interleukin-1β, interleukin-2, interleukin-2 receptor, interleukin-4, interleukin-10, interleukin-18, interferon-γ, C-reactive protein, granulocyte colony-stimulating factor, interferon-γ, CXCL10, monocyte chemoattractant protein 1, macrophage inflammatory protein 1-α, and tumor necrosis factor-α [10, 12]. Concomitantly, most patients show signs of T cell exhaustion with lower lymphocyte counts. The fact that systemic inflammation has been shown to promote cognitive decline and neurodegenerative disease makes it likely that COVID-19 survivors will experience neurodegeneration in the following years [13, 14]. Of note, cytokine levels can predict the subsequent occurrence of hippocampal atrophy in patients that experience severe sepsis [15]. In keeping with this, the most frequent clinical presentation of COVID-19 is the development of acute respiratory distress syndrome (ARDS) [16], the latter being, along with chronic ventilation, highly associated with subsequent cognitive decline, executive dysfunction, and reduced quality of life, often persisting months and years after hospital discharge [17] reviewed in [18]. The causative role of systemic inflammatory damage to the brain is further supported by the fact that none of the cerebrospinal fluid samples investigated in the study by Helms et al. found evidence for a direct presence of SARS-CoV-2 in the cerebrospinal fluid [2]. That being said, one should not ignore the potential importance of the single case report of direct viral infiltration of the brain and viral encephalitis, either by hematogenous or neuronal retrograde dissemination [19].

Evidence from murine lung injury models and ARDS patient samples emphasize the role of the NLRP3 inflammasome in the pathogenesis and detrimental outcome of ARDS [20, 21]. In keeping with this, the coronavirus ORF3a protein has been shown to induce NLRP3 inflammasome activity [22]. Moreover, ventilation-induced hypercapnia has been experimentally shown to lead to cognitive impairment in a NLRP3 inflammasome-interleukin-1β-dependent manner [23]. Given the above cytokine findings in COVID-19 patients and in particular the rise of interleukin-1β and interleukin-18, it seems highly likely that COVID-19 patients suffer from NLRP3 inflammasome activation. This activation and the subsequent increased activity of proinflammatory immune pathways are likely to exert a negative impact on cerebral homeostasis and function.

This conclusion is based on not only epidemiological evidence but also on experiments that showed that systemic, NLRP3 inflammasome-mediated inflammation adversely affects beneficial immune functions in the brain and thereby causes the pathological accumulation of neurodegeneration-associated peptides such as fibrillar amyloid-β [24]. Thus, both peripheral and central induction of the NLRP3 inflammasome can directly induce or aggravate neurodegenerative processes that lead to functional impairment in AD [25] or strongly contribute to the spreading of pathology and thus the progression of the disease [26]. The recent finding showing that NLRP3-driven and interleukin-1β-mediated modulation of phosphokinases and phosphatases largely accounts for the pathological formation of neurofibrillary tangles in murine models of tauopathy raises the concern that COVID-19 patients are likely to experience an induction or strong aggravation of neurodegenerative processes [27].

## Conclusion

Evidence strongly suggests that patients surviving COVID-19 are at high risk for subsequent development of neurological disease and in particular Alzheimer’s disease. Neurologists, psychiatrists, and caregivers should be alerted to a possible increase in such cases in COVID-19 survivors. Prospective studies are needed to investigate potential correlations between acute and sub-acute COVID-19 infections and long-term neurological sequalae in this patient cohort.

## Data Availability

Not applicable.

## Abbreviations

AD: Alzheimer’s disease
ARDS: Acute respiratory distress syndrome
COVID-19: Coronavirus SARS-CoV-2
CSF: Cerebrospinal fluid
NLRP3: NOD-, LRR-, and pyrin domain-containing protein 3
ORF3a: Not characterized yet (UNIPROT)
